# E-learning in health professions education during the COVID-19 pandemic: a systematic review

**DOI:** 10.3352/jeehp.2021.18.27

**Published:** 2021-10-29

**Authors:** Aziz Naciri, Mohamed Radid, Ahmed Kharbach, Ghizlane Chemsi

**Affiliations:** 1 Multidisciplinary Laboratory in Sciences and Information, Communication and Education Technology, Faculty of Sciences Ben M’Sik, Hassan II University of Casablanca, Casablanca, Morocco; 2 Laboratory of Physical Chemistry of Materials, Faculty of Sciences Ben M’Sik, Hassan II University of Casablanca, Casablanca, Morocco; 3 Observatory of Research in Interdisciplinary Didactics and University Pedagogy, Faculty of Sciences Ben M’Sik, Hassan II University of Casablanca, Casablanca, Morocco; 4 Laboratory of Biostatistics, Clinical Research and Epidemiology, Faculty of Medicine and Pharmacy of Rabat, Mohamed V University, Rabat, Morocco; 5 High Institute of Nursing Professions and Technical Health, Agadir, Morocco; Hallym University, Korea

**Keywords:** Computer-assisted instruction, COVID-19, Distance education, Health occupations, Morocco

## Abstract

As an alternative to traditional teaching, e-learning has enabled continuity of learning for health professions students during the coronavirus disease 2019 (COVID-19) pandemic. This review explored health professions students; perceptions, acceptance, motivation, and engagement with e-learning during the COVID-19 pandemic. Following the Preferred Reporting Items for Systematic Reviews and Meta-Analyses guidelines, a systematic review was conducted by consulting 5 databases: PubMed, ERIC (Ebsco), Science Direct, Scopus, and Web of Science. The quality of the included studies was assessed using the Medical Education Research Study Quality Instrument. The research protocol was previously registered in the PROSPERO registry (CRD42021237055). From 250 studies identified, 15 were selected with a total of 111,622 students. Mostly positive perceptions were reported in 7 of 12 studies, which mainly focused on technology access, possession of basic computer skills, pedagogical design of online courses, online interactions, and learning flexibility. However, predominantly negative perceptions were identified in 5 of 12 studies, which pointed out constraints related to internet connections, the use of educational platforms, and acquisition of clinical skills. Satisfactory levels of acceptance of distance learning were reported in 3 of 4 studies. For student motivation and engagement, 1 study reported similar or higher motivation than with traditional teaching, and another study indicated that student engagement significantly increased during the COVID-19 pandemic. Health professions students showed a positive response to e-learning regarding perceptions, acceptance, motivation, and engagement. Future research is needed to remediate the lack of studies addressing health professions students’ motivation and engagement during the COVID-19 pandemic.

## Introduction

### Rationale

On March 11, 2020, the World Health Organization declared coronavirus disease 2019 (COVID-19) to be a pandemic disease [1]. This urgent and unexpected situation has caused unprecedented challenges to health systems, especially in the health sciences education sector, where concerns have been raised regarding the possibility that medical and nursing students could contract COVID-19 during their training and become potential transmitters of the virus in health care institutions. In the context of this crisis, changes in the teaching-learning paradigm have become an unavoidable necessity [2]. E-learning is an important alternative to ensure continuity of learning while protecting students against the risk of COVID-19 infection in the campus environment [3]. Indeed, e-learning is a teaching approach based on digital media and devices as tools to improve access to training, communication, and interactions between teachers and students [4]. It is a relatively new and growing approach in health professions education [5]. This pedagogical model offers a flexible learning environment where students can learn at their own pace without time or space constraints through various educational content (text, image, audio, and video). When using e-learning platforms, students interact with teachers, educational content, and technological innovations [6].

However, the sudden transition to distance learning during COVID-19, combined with the concomitant time constraints, prevented the effective implementation of e-learning [5]. Furthermore, some teachers had limited skills in e-learning-based pedagogical methods [7]. A lack of motivation and absence of interactions between teachers and students were also pointed out as the main disadvantages [8].

### Objectives

The purpose of this review was to explore health science students’ perceptions, acceptance, motivation, and engagement with e-learning during the COVID-19 pandemic. The specific research questions were as follows: (1) What are students’ perceptions of the implementation of e-learning during the COVID-19 pandemic? (2) Did heath profession students accept the adoption of e-learning during COVID-19? (3) What is the motivational level of health professions students towards e-learning during the COVID-19 crisis? (4) What is the engagement level of students during the transition to e-learning during COVID-19?

## Methods

### Ethics statement

This was not a human-subject study; therefore, neither approval by the institutional review board nor obtainment of the informed consent was required.

### Registration and protocol

This systematic literature review was conducted following the Preferred Reporting Items for Systematic Reviews and Meta-Analyses (PRISMA) guidelines [9]. The research protocol was previously registered in the International Prospective Register of Systematic Reviews (PROSPERO: CRD42021237055; available from: https://www.crd.york.ac.uk/prospero/display_record.php?ID=CRD42021237055).

### Eligibility criteria

Inclusion and exclusion criteria were developed using the population, intervention, comparison, outcome, and study design (PICOS) framework (Table 1).

### Information sources

PubMed, ERIC (Ebsco), Science Direct, Scopus, and Web of Science databases were checked for English-language studies published from March 11, 2020, to February 10, 2021. The databases were last accessed on February 11, 2021.

### Search strategy

The database searches (PubMed, ERIC [Ebsco], Science Direct, Scopus, and Web of Science) were conducted independently by 2 authors (A.N., A.K.), using the following keywords: (e-Learning) or (online learning) or (distance learning) or (distance education) and (medical education) or (nurs* education) and (COVID-19) or (2019-novel coronavirus) or (2019-nCoV) or (SARS-CoV-2). Examples of the search terms are as follows:

**Scopus:**
(TITLE-ABS (e-learning OR "online learning" OR "distance learning" OR "distance education")) AND (TITLE-ABS ("medical education" OR "nurs* education")) AND (TITLE-ABS (covid-19 OR "2019-novel coronavirus" OR 2019-ncov OR sars-cov-2))
**PubMed:**
((e-Learning[Title/Abstract] OR "online learning"[Title/Abstract] OR "distance learning"[Title/Abstract] OR "distance education"[Title/Abstract]) AND ("medical education" [Title/Abstract] OR "nursing education"[Title/Abstract])) AND (covid-19 [Title/Abstract] OR "2019-novel coronavirus" [Title/Abstract] OR 2019-ncov [Title/Abstract] OR sars-cov-2[Title/Abstract]).

### Selection process

Two authors (A.N. and A.K.) eliminated duplicate articles using EndNote X9 (Clarivate, Philadelphia, PA, USA). They independently checked titles and abstracts to identify potentially included studies. This selection and filtering process was performed using Rayyan QCRI (Rayyan Systems Inc., Cambridge, MA, USA), a web and mobile application for systematic reviews [10]. In cases of persistent disagreement between the 2 authors, a third reviewer (G.C.) was requested to make a final decision. The full texts of the included articles were downloaded for further evaluation, and the reference lists of all relevant articles were reviewed to identify additional literature.

### Data collection process

One author (A.N.) collected the data from the selected studies using an extraction form prepared in consensus between the authors. Another author (A.K.) verified the collected information. In case of any disagreement, a third author was consulted for a final decision.

### Data items

Data were extracted on the following general characteristics of the selected studies: authors, year, country of provenance, study design, participants’ characteristics (discipline, age, gender), and educational platforms used. Moreover, data on students’ perception, acceptance, motivation, and engagement (mean or median scores or effective/percentage) towards e-learning were extracted. The measurement instruments used (items and domains) were also selected.

### Study risk of bias assessment

The risk of bias assessment of the included studies was conducted independently by 2 authors (A.N. and M.R.) using the Medical Education Research Study Quality Instrument (MERSQI). The MERSQI is a tool for assessing the methodological quality of quantitative research articles. The scale consists of 10 items organized into 6 domains: study design, sampling, data types, validity of the assessment instrument, data analysis, and outcomes. The total score ranges from 5 to 18. The agreement between the 2 examiners’ results was analyzed using the kappa statistical coefficient (κ).

### Synthesis methods

The data were classified and analyzed to achieve the objectives of the review. Students’ perceptions were extracted and tabulated with key items such as technology access, possession of basic computer skills, instructional design of online courses, online interactions, learning flexibility, health issues, and acquisition of clinical knowledge during online learning. Data synthesis was initially conducted by the first author (A.N.) and then was discussed with 2 other authors (A.K. and G.C.). The included studies were analyzed through a narrative synthesis. Due to the disparity and heterogeneity among studies’ results, a meta-analysis was not performed.

### Reporting bias assessment

Reporting bias in this systematic review was assessed independently by 2 authors (A.N. and A.K.) in terms of selective outcome reporting by comparing study results with previously published study protocols and registrations. Any disagreement was resolved by consulting the opinion of a third author (G.C.).

### Certainty assessment

Not done.

## Results

### Study selection

**Fig. 1.**
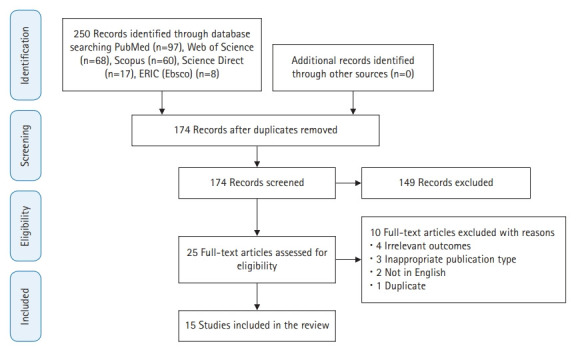
Study selection process according to the PRISMA (Preferred Reporting Items for Systematic Reviews and Meta-Analyses) diagram.

The search strategy identified a total of 250 articles, of which 76 were duplicates. Of the 174 studies that were screened by title and abstract, 149 were excluded. The 25 studies that were eventually eligible were downloaded for a full review. Finally, 15 studies were included in this review [11-25]. The PRISMA flow chart illustrates the selection process of the included studies (Fig. 1).

### Study characteristics

**Fig. 2.**
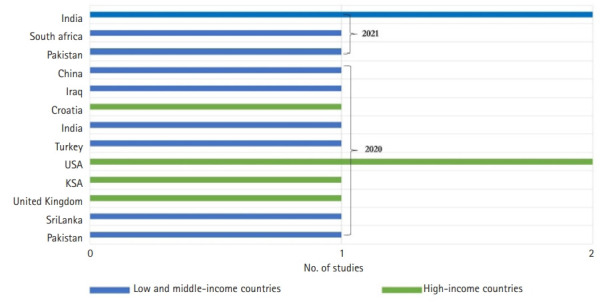
Year of publication, country of origin of included studies and country classification.

Fifteen studies were considered eligible for this review. One-third of the studies were conducted in high-income countries [15,17,18,21,23], and two-thirds in low- and middle-income countries [11-14,16,19,20,22,24,25] (Fig. 2). The total number of participants was 111,622. The sample size of the studies ranged from 30 [18,23] to 99,559 students [25]. A total of 106,152 participants (95.1%) were medical students, as reported in 10 studies [14-20,23-25]. One study included 60 nursing students [13]. In other studies, the focus was on heterogeneous samples in terms of discipline. Two studies involved 4,745 medical and nursing students [21,22], while 2 other investigations sampled 665 medical and dental students [11,12]. The gender of participants was reported in 8 studies [11-13,15,17,22,23,25]. The gender ratio of the included studies ranged from 0.3 [23] to 0.71 [13]. Almost half of the studies were conducted among undergraduate students [12-16,19,22].

All studies adopted fully online courses. The teaching platforms used were explicitly indicated in 8 studies [13,14,17-20,23,24]. Moodle was used in 2 studies [13,19], Zoom in 2 other studies [18,20], and WebEx video conferencing in 1 study investigation [23]. One study used both Zoom and Facebook as a freely accessible social media platform [14], and another opted for Zoom and Blackboard [17]. Google Classroom and Free Conference Call software were used in another study [24].

Students’ perceptions were investigated by 9 studies [11-13,16,18,20,23-25] and acceptance by 3 studies [14,19,22]. In addition, 1 investigation tested students’ perceptions and motivation [21], 1 study tested perceptions and acceptance [17], and 1 article discussed students’ perceptions and engagement [15] (Table 2).

### Risk of bias in studies

The methodological quality of half of the included studies as assessed by the MERSQI scale was relatively moderate, with a mean score of 8.50±1.44 and a median score of 8.50 (interquartile range, 7.5–9.5). The MERSQI score of the studies ranged from 6 [14] to 11.5 points [25], out of a total of 18. The kappa coefficient of concordance was 0.77. All included studies were cross-sectional studies with no control group, and participants were limited to fully online learning. The majority of studies used questionnaires developed by the authors. Furthermore, the response rate was less than 50% in 9 studies [11-17,20,24], and ranged from 50% to 74% in 6 studies [18,19,21-23,25]. The data analysis in 6 studies was limited to a descriptive level [14,16,18,21,23,24] (Table 3).

### Results of individual studies

Relevant data from the included studies are grouped and summarized separately in Table 2 and Supplement 1.

### Results of syntheses

#### Students’ perceptions

Twelve of the 15 included studies examined students’ perceptions of e-learning during the COVID-19 pandemic, either in isolation or in addition to another parameter [11-13,15-18,20,21,23-25]. The measurement tools were developed principally by the authors, which led to different results due to heterogeneity in the items. The items were mainly related to major aspects of e-learning (technology access, possession of basic computer skills, pedagogical design of online courses, online interactions, learning flexibility, health issues, and acquisition of clinical knowledge during online learning). Positive perceptions of e-learning were predominantly recorded in 7 studies, including a total of 3,863 students [12,13,17,18,20,21,23]. Four studies (57.2%) were conducted among medical students [17,18,20,23], 1 among medical and dental students [12], 1 among medical and nursing students [21], and 1 exclusively among nursing students [13]. Four studies were conducted in high-income countries [17,18,21,23].

A study by Kumar et al. [20] in 2020 compared students’ perceptions of a synchronous and an asynchronous course. Students’ perception scores were better for the direct classroom course than the online sessions (47.5±2.61 versus 35.5±3.25, P<0.001) [20]. Otherwise, the positive perceptions described by most students in other studies were related to technology access, possession of computer skills, online course design, online interactions and learning flexibility. Positive perceptions were reported for access to technological equipment in 3 studies (76.7% to 86%) [12,21,23], the possession of basic computer skills in 3 studies (51.2% to 84.7%) [12,17,21] and the instructional design of online courses in 6 studies, including an attractive learning content in 4 studies (48.8% to 84.2%) [12,13,17,21] and video learning in 3 investigations (58.1% to 89.7%) [12,18,21]. Moreover, 3 studies revealed positive perceptions of interactions on the platform (69.5% to 86.7%) [17,21,23] and learning flexibility in 4 studies (52.3% to 100%) [12,17,18,23]. Otherwise, mainly positive perceptions of e-learning were mentioned in 5 studies [11,15,16,24,25]. Four of these studies were conducted among medical students [15,16,24,25], and 1 study was conducted among medical and dental students [11]. Most of these studies (80%) were performed in low- and middle-income countries [11,16,24,25].

Negative perceptions in the included studies focused on access to the internet in 2 studies (21.5% to 35.9%) [15,16], health problems caused by the use of e-learning in 2 studies (62.5% to 67%) [18,24], and difficulty in developing clinical skills online in 2 studies (82.2% to 84.2%) [15,17]. The results are presented in more detail in Supplement 1.

#### Students’ acceptance

Among the 15 included studies, students’ acceptance of e-learning was investigated in 4 studies, with a total sample size of 4,553 participants [14,17,19,22]. Three (75%) of these 4 studies were conducted among medical students exclusively [14,17,19], and 1 was conducted among medical and nursing students [22]. Three-quarters of these studies were conducted in low- and middle-income countries [14,19,22].

Singh et al. [22] in 2021 approached e-learning acceptance by exploring 4 components. The first was related to the feasibility/practicality of online courses, including internet connectivity, device logistics, internet and computer literacy, and the availability of a dedicated space for participating in online classes. The second was associated with health issues during online courses. The third was about online teaching methods, including the type of teaching methods and class time allocation. The fourth dealt with students’ attitudes towards e-learning and their preferences. In this context, Singh et al. [22] in 2021 reported that 18.2% of students had a personal desktop/laptop computer, and those from affluent families and those living in cities had better access to e-learning facilities (internet connectivity, availability of a personal computer, a dedicated room, and training in computer/internet use). PowerPoint presentations were the most frequently used teaching method (80%). Loss of concentration (58.1%), eye strain (54%), and sleep disturbance (42.8%) were the most common health problems among students who attended classes for more than 4 hours per day. Moreover, there was insufficient interaction time with the instructor (30%). Regarding students’ attitudes and preferences towards e-learning, less than a quarter (20.4%) of the participants felt that online learning could replace traditional teaching (15% of medical students versus 30% of nursing students, P=0.001). Students expressed preferences for 3–6 classes per day, with each lasting for <40 minutes and a 10- to 20-minute break between classes and/or interactive sessions [22].

Two other studies reported moderate levels of e-learning acceptance [17,19]. The study conducted by Ibrahim et al. [17] in 2020 reported a mean score of 102.82±24.10 (min: 21, max: 147) using the E-Learning Acceptance Measure, which was composed of 3 components: tutor quality (with a mean score of 39.34±10.14), perceived usefulness (with a mean score of 44.11±11.52), and facilitating conditions (with a mean score of 19.36±5.85). Moreover, a second investigation conducted by Kolcu et al. [19] in 2020 highlighted a total score of 56.99% using the Learning Management System Acceptance Scale, which contained 4 sub-dimensions: performance expectancy (56.75%), effort expectancy (62.00%), facilitating conditions (59.68%), and social influence (44.67%).

Similarly, Chandrasinghe et al. [14] in 2020 reported very satisfactory levels of acceptance regarding the relevance of the topics studied (90%), the importance of the topics (89%), interactions within the platform (87.1%), the improvement of interest in clinical medicine (79.3%), the acquisition of skills and understanding of topics (88%), and the importance of the topics covered in examinations and clinical practice (90%).

#### Students’ motivation

Motivation in a distance learning environment during the COVID-19 pandemic was tested in 1 study [21]. This study was conducted at 9 health science institutions with 2,520 medical and nursing students. Student motivation was measured by a questionnaire developed by the authors with 2 items. The first item focused on motivation to participate in online learning compared to classroom courses, and 64.4% of participants demonstrated an equal or higher motivational level to attend exclusive e-learning. The second item related to student motivation during the progression of online courses, and more than half of participants (65.5%) reported an equal or higher level of motivation to participate in a longer duration of exclusive e-learning.

#### Students’ engagement

Student engagement was reported in 1 study that was conducted among 2,721 medical students [15]. Student engagement was measured by a questionnaire developed by the authors through an analysis of the number of hours spent on e-learning during the COVID-19 pandemic compared to traditional teaching before the pandemic. Students spent an average of 7–10 hours using e-learning platforms during the pandemic, compared to 4–6 hours before the pandemic (P<0.05). Similarly, the number of students spending more extended periods on online teaching platforms significantly increased during the COVID-19 health crisis. Furthermore, small group work methods, group discussions, online case simulations, and quizzes helped increase student engagement [15].

### Reporting biases

The search showed that no protocols or records of the included studies were previously published. The risk of reporting bias was unclear because it was not possible to determine whether all the results were included in the published reports.

## Discussion

### Summary of evidence

This systematic review explored the different aspects of health science students’ perceptions, acceptance, motivation, and engagement with e-learning during the COVID-19 pandemic. The majority of studies reported positive perceptions of e-learning. These results are similar to the findings of literature reviews conducted before and during the COVID-19 pandemic [26-28]. However, Xhelili et al. [29] in 2021 found positive perceptions of traditional learning during the COVID-19 pandemic by Albanian university students. The participants in that study reported difficulties related principally to the unavailability of internet connection and the lack of technological devices.

In our review, the positive trend in perceptions of distance learning may be due primarily to the sudden and unexpected shift to e-learning, which generated a sense of security among students during the spread of the virus. This situation probably caused a forced adaptation to the online learning requirements to ensure continuity of learning during these exceptional times. Second, positive perceptions could also be explained by remarkable advances in recent years in computer-based platforms in health sciences education, which have progressed to the point that they offer a learning climate similar to face-to-face teaching. Furthermore, technology access, computer skills, pedagogical engineering quality of online courses, and learning flexibility were the main items associated with positive perceptions of e-learning. Nevertheless, limited internet connection, technical problems in using educational platforms, and difficulties in acquiring clinical skills were the most important constraints and limitations reported by students in online learning. Consistent findings have been reported in other studies conducted during the pandemic [29-32].

Satisfactory levels of e-learning acceptance were expressed by health professions students during the COVID-19 pandemic. Other studies supported this finding [33,34]. Nevertheless, a previous study conducted by Ngampornchai and Adams [35] in 2016, using the unified theory of acceptance and use of technology, reported that students had a cautious tendency to accept online learning. Performance expectancy and effort expectancy were strong indicators of technology acceptance. The predisposition to accept distance learning courses in the current study could be attributed, on the one hand, to the usability of e-learning platforms. On the other hand, the concordance between the educational content of health sciences training and didactic conceptions of courses (video learning, serious games) may be a contributor. Taat and Francis [33] in 2020 showed that usability and ease of use platforms, lecturer characteristics, system quality, the information provided, and available technical support were the main factors influencing the acceptance of e-learning.

With regard to students’ motivation, our review found an equal or higher motivation to attend exclusive e-learning compared to classroom learning. This result is in agreement with previous studies’ findings [36-38]. However, Aguilera-Hermida et al. [39] in 2021 demonstrated a decrease in students’ motivational levels in Mexico, Peru, and the United States after switching to e-learning during the COVID-19 pandemic and a significantly unchanged motivational level in Turkish students. This study also reported that lower motivation in students in the United States, Peru, and Mexico during online learning was associated with worse cognitive engagement. In the current review, the significant observed improvement in motivation in a digital environment could be related to the temporospatial flexibility of the pedagogical content, accessibility, and technological skills [40].

Regarding student engagement, the only study included that investigated this parameter showed a statistically significant difference between the number of hours spent on e-learning before and during the pandemic. This result is corroborated by the exclusive and massive adoption of e-learning during the confinement and post-confinement periods of several waves of outbreaks of COVID-19 variants. This finding of our review contradicts those of other studies conducted during the COVID-19 pandemic [41,42]. Wester et al. [41] in 2021 reported a significant decrease in the overall engagement score calculated from students’ behavioral, cognitive, and emotional engagement scores during the pandemic. Moreover, Chan et al. [42] in 2021 found that 55% of nursing students were not highly engaged in an online course during the COVID-19 pandemic. Psychological motivation, peer collaboration, cognitive problem solving, interaction with instructors, community support, and learning management are factors that can improve student engagement in a digital environment [43].

### Limitations

This review has some limitations. First, all included studies were cross-sectional. Second, the authors used various measurement instruments, and the consequent disparity among items made an integrative analysis difficult. Third, student motivation and engagement were measured in the included studies through 2 items for each. In addition, a certainty assessment was not performed. Therefore, we must be cautious in interpreting the results. The last limitation is that non-English-language articles were not reviewed.

### Implications for practice and future research

The results of this review encourage decision-makers in the health professions education field to integrate e-learning into training programs by ensuring, on the one hand, equitable access to technological equipment and internet connections and, on the other hand, developing students’ computer skills. Furthermore, pedagogical approaches based on digital teaching could be beneficial as an alternative or complement to traditional teaching. If this method had been adopted before in health professions education, institutions could have more easily overcome the unprecedented challenges caused by the emergence of this COVID-19 crisis. Therefore, investment in the implementation of e-learning remains an unavoidable necessity because the return on investment could be decisive in terms of the quality of training received by health science students and consequently to the health services provided to the population. Moreover, the data explored in this study showed a lack of evidence, especially for students’ motivation and engagement with e-learning during the COVID-19 pandemic. Further methodologically rigorous research is needed, especially in the Middle East/North Africa region, to help fill this gap. It is also essential to investigate the problems of failure and dropout in health professions education in relation to online learning during this pandemic.

## Conclusion

Switching to online learning to ensure the continuity of learning during the COVID-19 pandemic was challenging for health professions education programs. The findings of this study indicate that the emergency shift to online health science learning during this health crisis received positive feedback from students in terms of perceptions, acceptance, motivation, and engagement. Although the socio-economic contexts of the countries differed, online learning was consistently facilitated by some major elements such as technology access, possession of basic computer skills, pedagogical design of online courses, and learning flexibility. In contrast, students reported constraints including access to internet connections, the use of educational platforms, and the acquisition of online clinical skills. These results should be noted in order to integrate these devices better into educational programs.

## Figures and Tables

**Table 1.** Eligibility criteria according to the PICOS framework

PICOS items	Inclusion and exclusion criteria
Population	Medical and dental students, nursing and health science students.
Intervention	Studies that examined fully online learning as a teaching method (synchronous and/or asynchronous). Studies that investigated hybrid teaching methods were excluded.
Comparison	Studies with or without a comparison group.
Outcome	Studies to be eligible for inclusion should include student perceptions, and/or acceptance, and/or motivation, and/or engagement in e-learning during the coronavirus disease 2019 pandemic.
Study design	We included cross-sectional studies, case-control studies, cohort studies, and randomized controlled trials. Qualitative studies, commentary articles, letters to the editor, editorials, conference abstracts, book chapters, and reviews were excluded.

PICOS, population, intervention, comparison, outcome, and study design.

**Table 2.** Summary of the characteristics of all included studies

Study (year)	Country	Study design	Discipline	No. of participants	Instructional design	Used platform	Results	Measurement instruments	Main domains and/or items of questionnaire
Abbasi et al. [11] (2020)	Pakistan	CSS	Medicine and dentistry	Medical students: 204	Fully online course	NR	P	23-item questionnaire, developed by the authors (5-point Likert scale)	Future learning preferences, comparison of e-learning with traditional teaching, quality and impact of e-learning, student-teacher interactions, online teaching security
				Dental students: 178					
Anwar et al. [12] (2021)	Pakistan	CSS	Medicine and dentistry	283	Fully online course	NR	P	20-item questionnaire, developed by the authors based on the original version of Watkins and his colleagues (5-point Likert scale)	Technology access, online skills and relationships, students’ views and students’ perceptions of possible advantages of e-learning
Buthelezi et al. [13] (2021)	South Africa	CSS	Nursing	60	Fully online course	Moodle	P	Questionnaire with 23 items, designed by the authors (4-point Likert scale)	Computer access, frequency of use, prior exposure to e-learning platforms, anxiety and attitude towards technology, perceived computer self-efficacy
Chandrasinghe et al. [14] (2020)	Sri Lanka	CSS	Medicine	1,047	Fully online course	Zoom, Facebook	A	Questionnaire designed by the authors	Relevance and importance of the topic, students’ discussions and clinical sense, discussion and knowledge and understanding of the topic
Dost et al. [15] (2020)	UK	CSS	Medicine	2,721	Fully online course	NR	P/E	20-item questionnaire, developed by the authors based in part on the Ready Education Environment Measure (5-point Likert scale)	The use and experience of online teaching during the COVID-19 pandemic, perceived benefits and barriers of online teaching
Gupta et al. [16] (2021)	India	CSS	Medicine	248	Fully online course	NR	P	22-item online survey developed by the authors (5-point Likert scale)	Time spent on online and offline learning, modality of didactic learning, interaction with educators, facilitating and hindering factors during online classes, comparison of e-learning with traditional teaching, the features preferred during online classes
Ibrahim et al. [17] (2020)	Saudi Arabia	CSS	Medicine	340	Fully online course	Zoom, Blackboard	P/A	21-item data collection sheets from the E-Learning Acceptance Measure and items to assess student perception (7-point Likert scale)	Preferred learning management system, tutor quality, perceived usefulness, facilitating conditions, perceptions regarding the benefits, enablers, and barriers to e-learning
Kivlehan et al. [18] (2020)	USA	CSS	Medicine	30	Fully online course	Zoom	P	Questionnaire developed by the authors	Perceived benefits and barriers to e-learning, satisfaction with education during the COVID-19 pandemic
Kolcu et al. [19] (2020)	Turkey	CSS	Medicine	941	Fully online course	Moodle	A	21-item questionnaire from the Learning Management System Acceptance Scale (5-point Likert scale)	Performance expectancy, effort expectancy, facilitating conditions, social influence
Kumar et al. [20] (2020)	India	CSS	Medicine	600	Fully online course	Zoom	P	10-item questionnaire developed by the authors (5-point Likert scale)	Flexibility of learning, achievement of pedagogical objectives, attractivity of the online course
Puljak et al. [21] (2020)	Croatia	CSS	Medicine and nursing	2,520	Fully online course	NR	P/M	73-item questionnaire developed by the authors (5-point Likert scale)	Personal experience with e-learning, motivation and attendance, possibility to participate in e-learning, continuation of education during the pandemic
Singh et al. [22] (2021)	India	CSS	Medicine and nursing	Medical student: 1,541	Fully online course	NR	A	33-item survey, developed by the authors (dichotomous and multiple-choice questions)	Feasibility of online classes, health issues arising from online classes, methods of online teaching, student preferences and attitudes towards e-learning
				Nursing student: 684					
Singhi et al. [23] (2020)	USA	CSS	Medicine	30	Fully online course	WebEx	P	19-item survey designed by the authors	Ease of technical access to the online platform, level of comfort with participation, knowledge acquisition, wellness, and COVID-19-specific coverage
Tuma et al. [24] (2020)	Iraq	CSS	Medicine	636	Fully online course	Google Classroom, Free Conference Call	P	10-item survey, designed by the authors	The feasibility of educational technology platforms for distance education and the education’s perceived quality
Wang et al. [25] (2020)	China	CSS	Medicine	99,559	Fully online course	NR	P	20-item questionnaire developed by the authors based in part on the technology acceptance model (5-point Likert scale)	Prior online learning experiences, perceptions of ongoing online education

CSS, cross-sectional study; NR, not reported; P, perception; A, acceptance; E, engagement; COVID-19, coronavirus disease 2019; M, motivation.

**Table 3.** Assessment of the methodological quality of included studies using the Medical Education Research Study Quality Instrument

Study	Study design	Sampling		Type of data	Validity of the evaluation instrument			Data analysis		Outcome				Total score
		Institution studied	Response rate score		Internal structure	content	Relationships to other variables	Appropriateness of analysis	Complexity of analysis	Satisfaction, attitudes, perceptions	Knowledge, skills	Behaviors	Patient/health care outcome	
Abbasi et al. [11]	1	0.5	0.5	1	1	1	0	1	2	1	0	0	0	9
Anwar et al. [12]	1	0.5	0.5	1	0	1	0	1	2	1	0	0	0	8
Buthelezi et al. [13]	1	0.5	0.5	1	0	1	1	1	2	1	0	0	0	9
Chandrasinghe et al. [14]	1	0.5	0.5	1	0	0	0	1	1	1	0	0	0	6
Dost et al. [15]	1	1.5	0.5	1	0	1	0	1	2	1	0	0	0	8
Gupta et al. [16]	1	0.5	0.5	1	0	1	0	1	1	1	0	0	0	7
Ibrahim et al. [17]	1	0.5	0.5	1	1	1	0	1	2	1	0	0	0	9
Kivlehan et al. [18]	1	0.5	1	1	0	1	0	1	1	1	0	0	0	7.5
Kolcu et al. [19]	1	0.5	1	1	1	1	0	1	2	1	0	0	0	9.5
Kumar et al. [20]	1	0.5	0.5	1	0	1	0	1	2	1	1.5	0	0	9.5
Puljak et al. [21]	1	1.5	1	1	0	1	0	1	1	1	0	0	0	8.5
Singh et al. [22]	1	1.5	1	1	0	1	1	1	2	1	0	0	0	10.5
Singhi et al. [23]	1	0.5	1	1	0	1	0	1	1	1	0	0	0	7.5
Tuma et al. [24]	1	0.5	0.5	1	0	1	0	1	1	1	0	0	0	7
Wang et al. [25]	1	1.5	1	1	1	1	1	1	2	1	0	0	0	11.5

## Supplementary materials

Data files are available from Harvard Dataverse: https://doi.org/10.7910/DVN/VFHJ1O

Supplement 1. Summary of the included studies' findings regarding students’ perceptions of the benefits and disadvantages of e-learning during the COVID-19 pandemic.

Supplement 2. Audio recording of the abstract.
